# Higher Serum BDNF Levels are Associated with Lower Risk of Cognitive Decline in Older Adults :The Otassha Study

**DOI:** 10.1093/geroni/igab046.2604

**Published:** 2021-12-17

**Authors:** Yoshinori Fujiwara, Kazushige Ihara, Mitsugu Hachisu, Hiroyuki Suzuki, Hisashi Kawai, Masahiro Hashizume, Hirohiko Hirano, Shuichi Obuchi

**Affiliations:** 1 Tokyo Metropolitan Institute of Gerontology, Tokyo, Tokyo, Japan; 2 Hirosaki University, Aomori, Alabama, Japan; 3 Showa University, Tokyo, Alabama, United States; 4 Toho University, Tokyo, Tokyo, Japan

## Abstract

Introduction: There has been growing interest in the use of circulating levels of brain-derived neurotrophic factor (BDNF) in the blood as a biomarker in the context of patients with Alzheimer’s and other neurodegenerative diseases. Prospective data on cognitive decline in the broad older population, however, remain limited. We assessed the relationship of serum BDNF levels with short-term decline in cognitive functioning of community-dwelling older adults. Methods. Prospective study of 405 adults 65-84 years old without dementia in Tokyo, Japan. The Montreal Cognitive Assessment-Japanese version (MoCA-J) and its subscales were used. Linear regression assessed standardized differences in test score differences between baseline (2011) and follow-up (2013) visits, according to baseline serum BDNF quartiles, with adjustment for baseline demographics, disease indicators, and cognitive scores. Results: Among participants who performed on the MoCA-J at baseline (scores in bottom quartile), cognitive decline was .65 (95% CI: .08 - 1.2; p=.025) standard deviations (SD) more pronounced in those with lowest than highest BDNF levels. Decline in executive function, but not in other subdomains, was also most pronounced in those with lowest baseline serum BDNF levels (difference: .32 SD; 95%CI: .08-.55; p=.007) Conclusion: Lower serum BDNF levels were associated with greater 2-year cognitive decline in community-dwelling older Japanese adults. Decline varied among cognitive subdomains, and baseline cognition. Research seeking to evaluate the added-value of serum BDNF for screening and/or health promotion initiatives involving physical activity, which has been linked to increment in BDNF levels, is warranted.

